# SUMOylation counteracts ubiquitination to stabilize ANKRD1 and promote osimertinib resistance in lung adenocarcinoma

**DOI:** 10.3389/fonc.2026.1960448

**Published:** 2026-10-22

**Authors:** BiaoFeng Fan, Qing Lei, HaoNan Wang, ChenJun Xin, XuanCheng Li, XiRui Zhu, JinXiang Kong, SiQi Xie, JianLiang Shang, ZuYang Li, Chao Deng, DongJin Lv, WeiWei Wang

**Affiliations:** 1 The Third Affiliated Hospital of Kunming Medical University, Yunnan Cancer Hospital, Yunnan Cancer Center, Kunming, China; 2 West China Hospital, West China School of Medicine, Sichuan University, Chengdu, China; 3 Haiyuan College of Kunming Medical University, Kunming, China

**Keywords:** ANKRD1, MERTK, osimertinib resistance, sumoylation, ZNF593

## Abstract

**Introduction:**

Acquired resistance to osimertinib remains a major challenge in the treatment of EGFR-mutant lung adenocarcinoma (LUAD), yet the contribution of stress-responsive protein homeostasis to resistance remains incompletely understood. This study aimed to investigate the role and regulatory mechanisms of ankyrin repeat domain 1 (ANKRD1) in acquired osimertinib resistance.

**Methods:**

Public transcriptomic datasets and machine learning-based feature selection were integrated with osimertinib-resistant LUAD cell models, paired clinical specimens, RNA sequencing, biochemical and molecular assays, functional rescue experiments, and in vivo tumor models to investigate the role of ANKRD1 and its upstream and downstream regulatory mechanisms in osimertinib resistance.

**Results:**

ANKRD1 was upregulated in osimertinib-resistant LUAD models and promoted tumor cell survival under therapeutic pressure. Mechanistically, zinc finger protein 593 (ZNF593) interacted with ANKRD1 and facilitated its SUMO1-dependent modification, which was associated with reduced ubiquitin-mediated degradation and enhanced ANKRD1 protein stability. Stabilized ANKRD1 subsequently promoted MERTK expression and sustained downstream AKT signaling, thereby supporting tumor cell survival under osimertinib pressure. Furthermore, K41 emerged as a critical regulatory residue, as K41R simultaneously impaired ANKRD1 SUMOylation, ubiquitination, and interaction with ZNF593, accompanied by attenuated downstream signaling and resistance phenotypes. These findings suggest that K41 plays an important role in coordinating ANKRD1 post-translational regulation and its interaction with ZNF593.

**Discussion:**

Our study identifies a proteostasis-related mechanism contributing to acquired osimertinib resistance and provides mechanistic insight into the ZNF593–ANKRD1–MERTK axis in EGFR-mutant LUAD. These findings support further investigation of this regulatory axis as a potential vulnerability in osimertinib-resistant LUAD.

## 1 Introduction

Targeted therapy has fundamentally reshaped the clinical management of epidermal growth factor receptor (EGFR)-mutant lung adenocarcinoma (LUAD), with the third-generation EGFR tyrosine kinase inhibitor (EGFR-TKI) osimertinib significantly prolonging patient survival (1–3). However, as with most targeted therapies, initial clinical responses are almost inevitably followed by the development of acquired resistance (4, 5). Established mechanisms include secondary EGFR alterations, bypass pathway activation, rare acquired oncogenic fusions such as CD74–ROS1, and phenotypic reprogramming (6–9). However, how upstream stress-adaptive processes coordinate protein homeostasis with signaling reprogramming during sustained therapeutic pressure remains incompletely understood.

Ankyrin repeat domain 1 (ANKRD1) is a stress-responsive protein originally characterized in the context of mechanical stress and cardiac remodeling (10, 11). Recent studies suggest that ANKRD1 exhibits considerable functional plasticity in cancer, exerting context-dependent roles across diverse tumor types and stress conditions (12, 13). These observations raise the possibility that ANKRD1 functions as an integrative mediator of cellular stress adaptation rather than a conventional oncogene or tumor suppressor.

Increasing evidence indicates that tumor drug resistance is governed not only by transcriptional reprogramming but also by post-translational modifications (PTMs) that regulate protein stability and signaling dynamics (14). Among these, SUMOylation and ubiquitination constitute a dynamic regulatory axis that fine-tunes protein degradation, subcellular localization, and the assembly of signaling complexes, particularly under stress conditions (15, 16). Notably, certain zinc finger proteins have been implicated in the regulation of post-translational modification processes, suggesting a potential role in integrating stress-adaptive signaling networks (17, 18). However, whether SUMOylation contributes to EGFR-TKI resistance—and more importantly, whether it functionally links nuclear regulatory processes to membrane receptor signaling—remains largely unexplored.

MER proto-oncogene, tyrosine kinase (MERTK), a member of the TAM receptor tyrosine kinase family, has been implicated in promoting tumor cell survival and therapeutic tolerance through activation of downstream AKT signaling (19, 20). Although the MERTK–AKT axis has been associated with resistance in multiple cancer types, its upstream regulatory mechanisms remain poorly defined, especially in the context of PTM-mediated control.

In this study, we investigate whether ANKRD1 contributes to osimertinib resistance through post-translational regulation and downstream receptor signaling. We show that ZNF593 interacts with ANKRD1 and facilitates its SUMO1-dependent modification, thereby supporting ANKRD1 protein stability. ANKRD1 subsequently promotes MERTK expression and AKT signaling. These findings identify a regulatory mechanism linking ANKRD1 proteostasis with MERTK–AKT signaling in osimertinib-resistant LUAD.

## 2 Materials and methods

### 2.1 Clinical sample collection

Clinical specimens were obtained from the biobank of Yunnan Cancer Hospital. Three pairs of matched tumor specimens were collected from patients with EGFR-mutant LUAD before osimertinib treatment and after the development of acquired osimertinib resistance. These paired specimens were used for exploratory immunohistochemical (IHC) evaluation of ANKRD1 expression rather than for a powered clinical cohort analysis. The study protocol was approved by the Ethics Committee of the Third Affiliated Hospital of Kunming Medical University, and all patients provided written informed consent. All procedures were conducted in accordance with the Declaration of Helsinki. Histopathological classification and staging were performed according to the 9th edition of the American Joint Committee on Cancer TNM staging system. All samples were independently evaluated by two experienced pathologists under blinded conditions to ensure diagnostic accuracy and consistency.

### 2.2 Dataset analysis

Transcriptomic datasets related to LUAD progression and acquired osimertinib resistance (GSE116959, GSE176348, GSE63459, GSE5281, and GSE5282) were retrieved from the Gene Expression Omnibus (GEO) database. Inclusion criteria included sufficient sample size, clear grouping information, and complete clinical annotations. Raw data were preprocessed and normalized using R software (v4.4.1), including background correction and normalization. Differential expression analysis was performed using the *limma* package, with thresholds set at |log2FC| > 1 and adjusted p < 0.05. Identified differentially expressed genes (DEGs) were subjected to subsequent machine learning-based feature selection.

### 2.3 RNA sequencing (RNA-seq) and bioinformatics analysis

Total RNA was extracted using TRIzol reagent (Thermo Fisher Scientific). RNA quality was assessed using a NanoDrop 2000 spectrophotometer and Agilent 2100 Bioanalyzer, ensuring RNA integrity number compliance. Library preparation and sequencing were conducted by Novogene using the Illumina NovaSeq 6000 platform (150 bp paired-end reads). Raw reads were processed using Trimmomatic for quality filtering, followed by alignment to the human reference genome (GRCh38) using HISAT2 (v2.2.1). Gene expression quantification was performed with featureCounts (v2.0.1), and differential expression analysis was conducted using DESeq2 (v1.30.1) with criteria |log2FC| > 1 and FDR < 0.05.

### 2.4 Cell culture and stable cell line generation

Human LUAD cell lines (HCC827, PC9) and HEK293T cells were obtained from the Chinese Academy of Sciences Cell Bank. All cells were routinely tested to ensure absence of mycoplasma contamination. Osimertinib-resistant cell lines (HCC827 OS, PC9 OS) were established through stepwise dose escalation over 4–6 months, culminating in stable proliferation in 100 nM osimertinib. Resistance was validated by CCK-8 assays and IC_50_ determination. Cells were maintained in DMEM (HEK293T) or RPMI-1640 (HCC827/PC9) supplemented with 10% FBS and 1% penicillin–streptomycin at 37 °C in 5% CO_2_. Lentiviral vectors for ANKRD1/MERTK overexpression and ANKRD1 shRNA/ZNF593 siRNA were constructed and transduced using standard protocols. Stable cell lines were selected using puromycin (2 μg/mL) or blasticidin (10 μg/mL).

### 2.5 Cell viability and drug sensitivity assay

Cells were seeded in 96-well plates (5 × 10^3^ cells/well) and treated with varying concentrations of osimertinib for 72 h. Cell viability was assessed using the CCK-8 assay, and IC_50_ values were calculated using nonlinear regression analysis.

### 2.6 Apoptosis assay

Apoptosis was quantified using Annexin V-FITC/PI staining followed by flow cytometry (BD FACSCalibur), allowing discrimination between early and late apoptotic cells.

### 2.7 EdU proliferation assay

Cell proliferation was evaluated using an EdU incorporation assay (Beyotime). Fluorescent images were acquired from five random fields and quantified using ImageJ.

### 2.8 Western blot analysis (WB)

Total protein was extracted using RIPA buffer supplemented with protease inhibitors and quantified using a BCA assay. Proteins were separated by SDS-PAGE and transferred onto PVDF membranes. Membranes were incubated with primary antibodies overnight at 4 °C, followed by HRP-conjugated secondary antibodies. Signals were detected using ECL reagents. All primary antibodies used in this study are listed in **Supplementary Table S1**.

### 2.9 Co-immunoprecipitation (Co-IP)

Cell lysates were incubated with specific antibodies or IgG controls, followed by Protein A/G magnetic beads. Immunocomplexes were eluted and analyzed by Western blot or subjected to mass spectrometry after silver staining.

### 2.10 Immunohistochemistry (IHC)

Paraffin-embedded sections underwent deparaffinization, antigen retrieval, and blocking before incubation with primary antibodies. Detection was performed using DAB staining, followed by hematoxylin counterstaining.

### 2.11 Immunofluorescence (IF)

Cells were fixed, permeabilized, and incubated with primary and fluorescent secondary antibodies. Nuclear staining was performed with DAPI, and images were acquired using confocal microscopy.

### 2.12 Proximity ligation assay (PLA)

The PLA was performed by using the Duolink *In Situ* Red PLA Kit (DUO92101; Sigma-Aldrich, USA). The cells were fixed and permeabilized as previously described for the immunofluorescence experiments. The samples were subsequently blocked and incubated overnight at 4 °C with two species-specific primary antibodies. PLUS and MINUS PLA probes diluted with antibody diluents, ligation buffer consisting of ligase and amplification solution containing polymerase were added according to the manufacturer’s instructions. The PLA signal was observed and imaged by using an Olympus FV3000 confocal fluorescence microscope (Olympus, Tokyo, Japan).

### 2.13 Protein stability assay (CHX chase)

Cells were treated with cycloheximide to inhibit protein synthesis, and protein degradation kinetics were assessed by Western blot.

### 2.14 Structural prediction

AlphaFold was used to predict the interaction interface between ANKRD1 and ZNF593, focusing on the spatial positioning of the K41 residue.

### 2.15 Xenograft model

Tumor xenografts were established by subcutaneous injection of 3 × 10^6^ cells into nude mice. Tumor volume was measured every 3 days using the formula: V = (L × W^2^)/2.

### 2.16 Orthotopic lung tumor model

Cells (1 × 10^6^) were injected into mouse lung tissue to establish orthotopic tumors. Osimertinib treatment was initiated 7 days after tumor implantation, and tumor burden was monitored by *in vivo* bioluminescence imaging.

### 2.17 Statistical analysis

Statistical analyses were performed using GraphPad Prism 9.0. Data are presented as mean ± SD. Differences were evaluated using Student’s t-test, chi-square test, or Spearman correlation analysis. A p-value < 0.05 was considered statistically significant.

## 3 Results

### 3.1 ANKRD1 is a convergent molecular determinant of LUAD progression and osimertinib resistance

Instead of focusing on individual candidate genes, we sought to identify upstream regulatory determinants that simultaneously govern LUAD progression and acquired resistance to osimertinib. Integrative transcriptomic analyses across multiple public datasets revealed distinct gene expression signatures associated with tumorigenesis and drug resistance (**Figures 1A, B**). Overlap of these signatures identified a subset of shared candidates (**Figure 1C**), suggesting the presence of common regulatory nodes.

**Figure 1:**
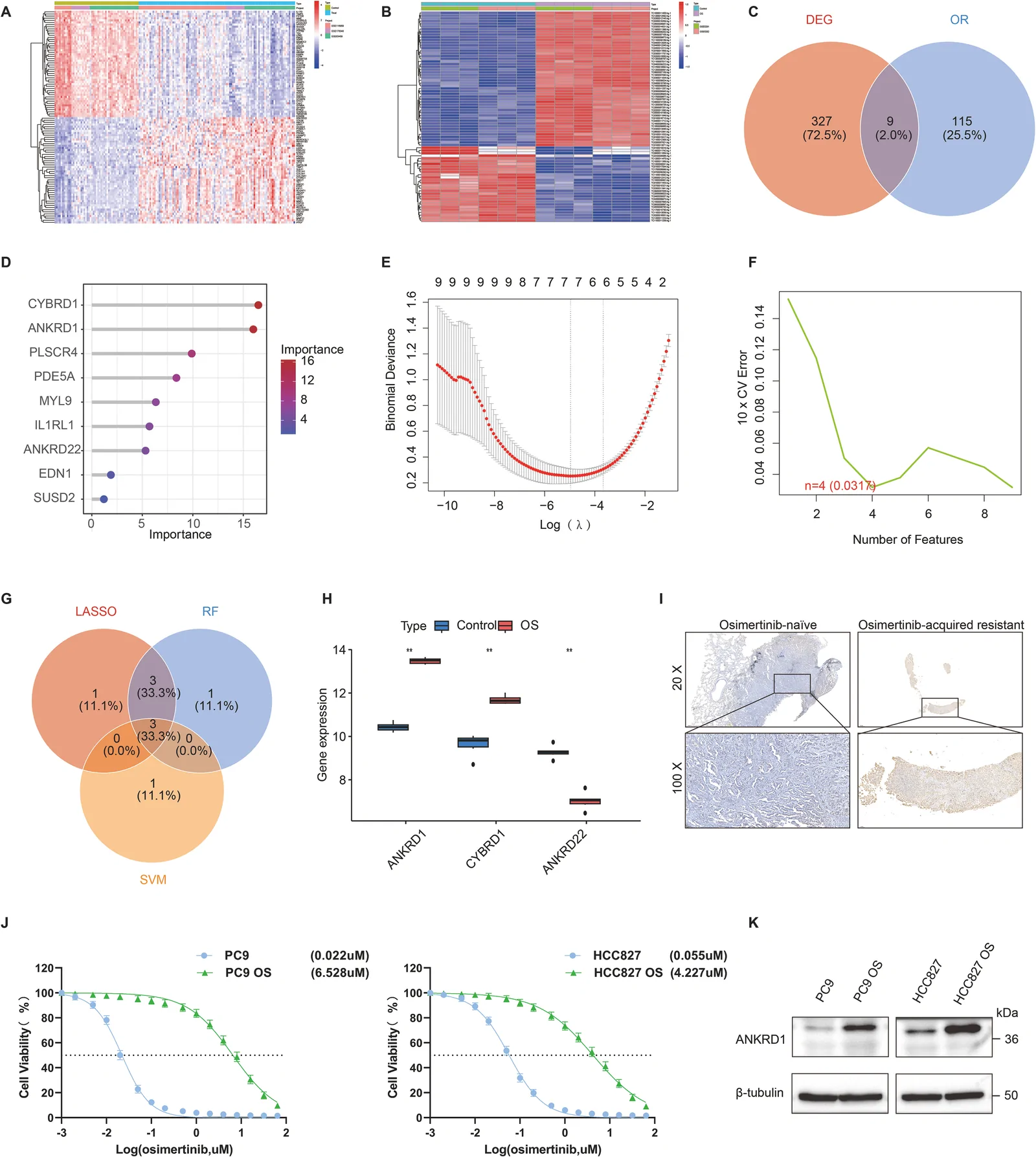
Identification of ANKRD1 as a key regulator in LUAD progression and osimertinib resistance. **(A)** Differential gene expression analysis between normal lung tissues and LUAD samples. **(B)** Differential expression analysis between osimertinib-naïve and -resistant LUAD samples. **(C)** Venn diagram showing overlapping genes between tumor-related and resistance-related datasets. **(D–F)** Feature selection using LASSO regression, random forest, and SVM-RFE models. **(G)** Intersection of candidate genes identified by three machine learning algorithms. **(H)** Expression pattern of ANKRD1 in LUAD tissues and resistant samples. **(I)** Representative IHC staining of ANKRD1 in three paired tumor specimens obtained before osimertinib treatment and after the development of acquired osimertinib resistance. **(J)** IC50 determination in parental and osimertinib-resistant PC9 and HCC827 cells. **(K)** Western blot analysis of ANKRD1 protein expression in resistant versus parental cells. Data are presented as mean ± SD from n = 3 independent experiments. Statistical significance was determined by Student’s *t*-test or one-way ANOVA as appropriate. *P* < 0.05 was considered statistically significant. **P < 0.01.

To further prioritize functionally relevant drivers, we applied a machine learning–based feature selection framework incorporating random forest, least absolute shrinkage and selection operator (LASSO) regression, and support vector machine–recursive feature elimination (SVM-RFE) (**Figures 1D–F**). This integrative strategy yielded three core genes (**Figure 1G**), among which ANKRD1 emerged as the most consistently upregulated factor in resistant contexts (**Figure 1H**).

IHC analysis of three paired clinical specimens showed consistently higher ANKRD1 expression after the development of acquired osimertinib resistance compared with the corresponding pre-treatment specimens (**Figure 1I**). Consistently, experimentally established resistant cell lines (PC9-OR and HCC827-OR) exhibited markedly increased IC_50_ values (**Figure 1J**) and elevated ANKRD1 protein levels (**Figure 1K**). These findings identify ANKRD1 as a convergent molecular determinant linking tumor progression and therapeutic resistance.

### 3.2 ANKRD1 functions as a stress-adaptive driver that sustains tumor survival under osimertinib pressure

To determine whether ANKRD1 is functionally required for resistance, we performed bidirectional perturbation experiments. While ANKRD1 overexpression in parental cells conferred reduced sensitivity to osimertinib, its depletion in resistant cells markedly restored drug responsiveness (**Figures 2A, B**; **Supplementary Figure S1**). Mechanistically, ANKRD1 knockdown significantly enhanced apoptosis under osimertinib treatment (**Figures 2C, D**) and suppressed proliferative capacity (**Figures 2E, F**), indicating a critical role in maintaining cell survival under therapeutic stress.

**Figure 2:**
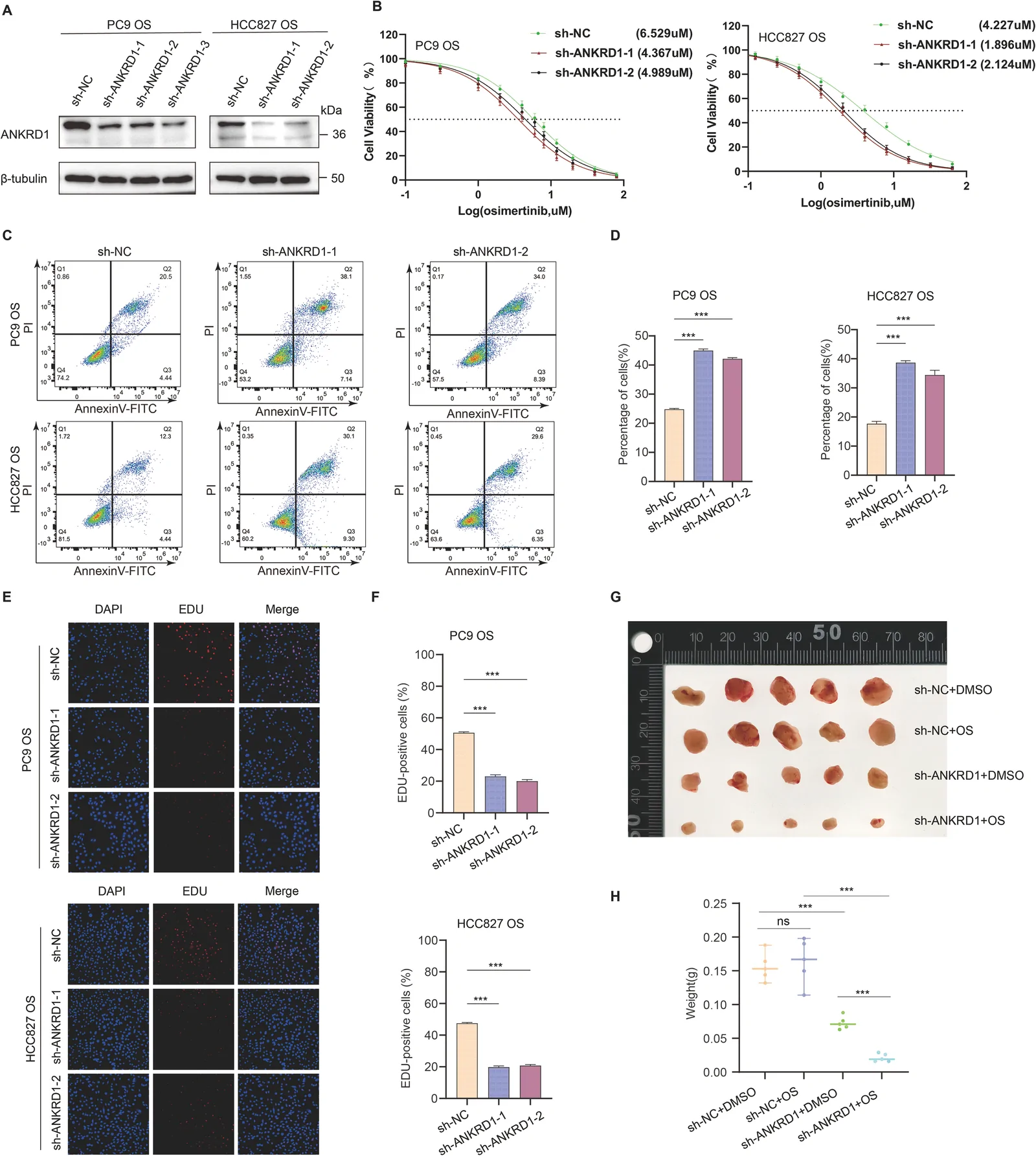
ANKRD1 promotes osimertinib resistance in LUAD cells. **(A)** Western blot validation of ANKRD1 knockdown efficiency. **(B)** Cell viability assays showing IC50 changes upon ANKRD1 modulation. **(C)** Representative flow cytometry plots of Annexin V/PI staining showing increased apoptosis following ANKRD1 knockdown under osimertinib treatment. **(D)** Quantification of apoptotic cells corresponding to **(C)**. **(E)** Representative images of cell proliferation assays following ANKRD1 depletion. **(F)** Quantification of cell proliferation corresponding to **(E)**. **(G)** Xenograft experiments showing that ANKRD1 depletion modestly affected tumor growth alone but enhanced the antitumor efficacy of osimertinib. **(H)** Quantification of tumor volume in xenograft models. Data are presented as mean ± SD from n = 3 independent experiments (*in vitro*) or n = 5 mice per group (*in vivo*). Statistical significance was determined by Student’s *t*-test or one-way ANOVA as appropriate. *P* < 0.05 was considered statistically significant. ***P < 0.001.

Importantly, *in vivo* xenograft models demonstrated that ANKRD1 depletion alone had a modest effect on tumor growth, but synergistically enhanced the antitumor efficacy of osimertinib (**Figures 2G, H**). This context-dependent phenotype suggests that ANKRD1 primarily functions as a stress-adaptive survival factor, rather than a classical oncogenic driver.

### 3.3 ANKRD1 rewires transcriptional output to activate the MERTK–AKT survival axis

To delineate the downstream program through which ANKRD1 promotes osimertinib resistance, we performed RNA-seq in ANKRD1-overexpressing cells and identified a distinct set of differentially expressed genes (**Figure 3A**). Integration of these genes with resistance-associated signatures further narrowed the candidate effectors linked to osimertinib resistance (**Figure 3B**). Among the overlapping candidates, MERTK emerged as a particularly relevant target because of its established role in receptor tyrosine kinase-mediated survival signaling and its functional connection to the PI3K/AKT pathway. We therefore focused on MERTK as a potential downstream mediator linking ANKRD1-dependent transcriptional regulation to the resistant phenotype. Consistent with this hypothesis, ANKRD1 expression positively correlated with MERTK expression (**Figure 3C**), and modulation of ANKRD1 altered MERTK expression, as further supported by qRT-PCR and dual-luciferase reporter assays (**Figures 3D, E**). In parallel, pathway enrichment analysis revealed enhanced PI3K/AKT signaling in ANKRD1-high cells (**Figure 3F** and **Supplementary Figure S1**), further supporting a functional connection between ANKRD1, MERTK, and downstream survival signaling.

**Figure 3:**
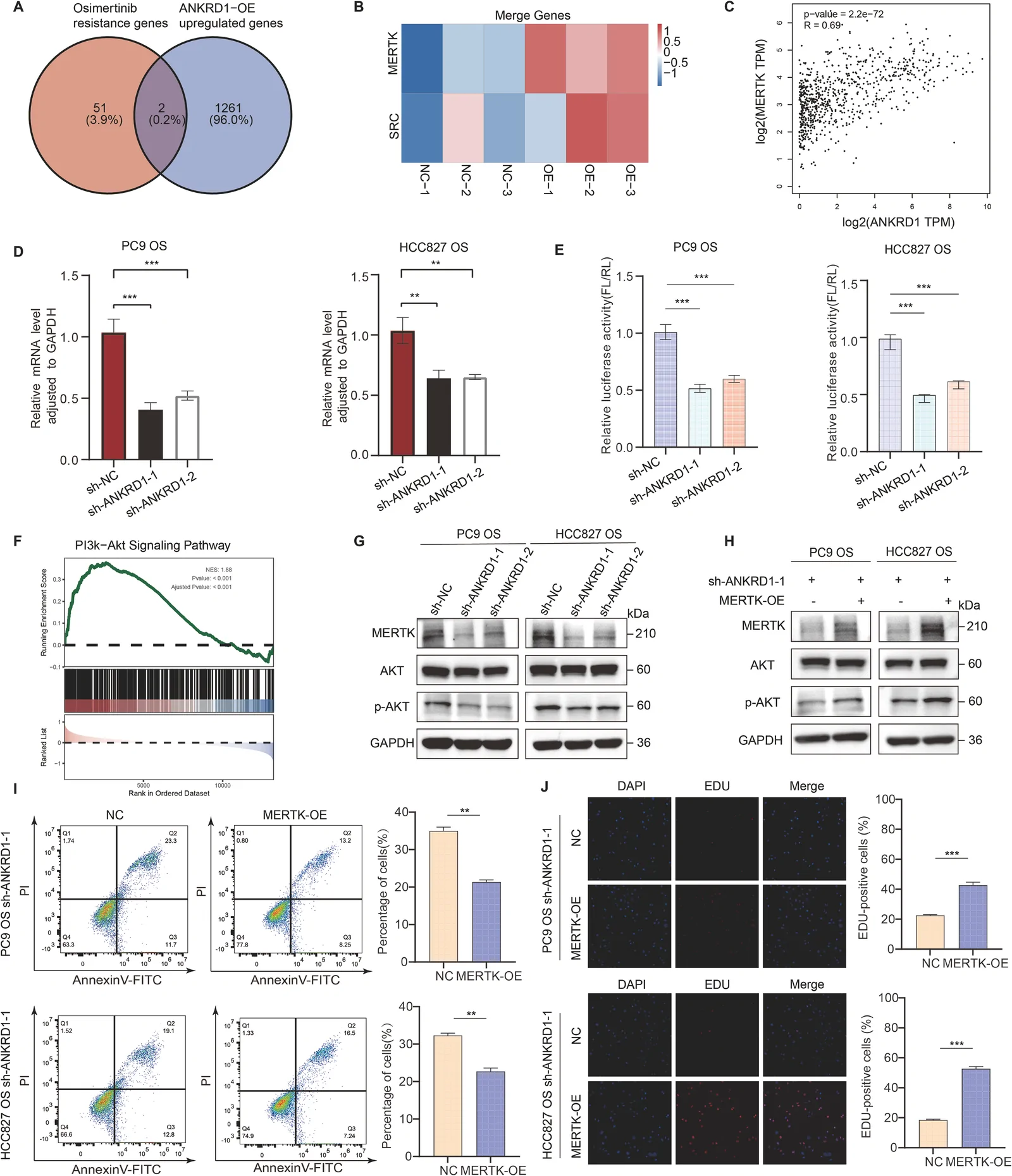
ANKRD1 activates the MERTK–AKT signaling pathway. **(A)** RNA-seq analysis showing differential gene expression profiles in ANKRD1-overexpressing cells. **(B)** Integration with resistance-associated gene sets identifying MERTK as a candidate downstream effector. **(C)** Correlation analysis between ANKRD1 and MERTK expression. **(D)** qRT-PCR validation of MERTK expression upon ANKRD1 knockdown. **(E)** Dual-luciferase reporter assay assessing ANKRD1-mediated transcriptional regulation. **(F)** GSEA plot showing enrichment of PI3K/AKT signaling in ANKRD1-high samples. **(G)** Western blot analysis of MERTK and AKT phosphorylation. **(H)** Rescue experiment showing restoration of AKT activation upon MERTK overexpression. **(I, J)** Functional assays showing effects of MERTK rescue on apoptosis and proliferation. Data are presented as mean ± SD from n = 3 independent experiments. Statistical significance was determined by Student’s *t*-test or one-way ANOVA as appropriate. *P* < 0.05 was considered statistically significant. **P < 0.01.

We next examined whether MERTK functionally mediates the effects of ANKRD1. ANKRD1 depletion reduced MERTK expression and AKT phosphorylation (**Figure 3G**), whereas re-expression of MERTK partially restored AKT activation and attenuated the increased apoptosis and impaired proliferation caused by ANKRD1 loss (**Figures 3H–J**). These rescue experiments identify MERTK–AKT signaling as an important downstream component of ANKRD1-mediated survival under osimertinib pressure.

### 3.4 ANKRD1 stability is governed by SUMO1-dependent proteostasis control

Given the stress-inducible nature of ANKRD1, we hypothesized that its regulation may occur at the level of protein homeostasis. Co-immunoprecipitation assays revealed that ANKRD1 undergoes selective SUMO1 modification, with no detectable modification by SUMO2/3/4 (**Figures 4A–C**). Functionally, SUMOylation inversely correlated with ubiquitination: resistant cells exhibited reduced ubiquitination of ANKRD1, whereas inhibition of SUMOylation markedly increased its ubiquitination (**Figure 4D**). These findings indicate that SUMOylation stabilizes ANKRD1 by antagonizing proteasomal degradation. This establishes ANKRD1 as a proteostasis-regulated node, rather than solely a transcriptional regulator.

**Figure 4:**
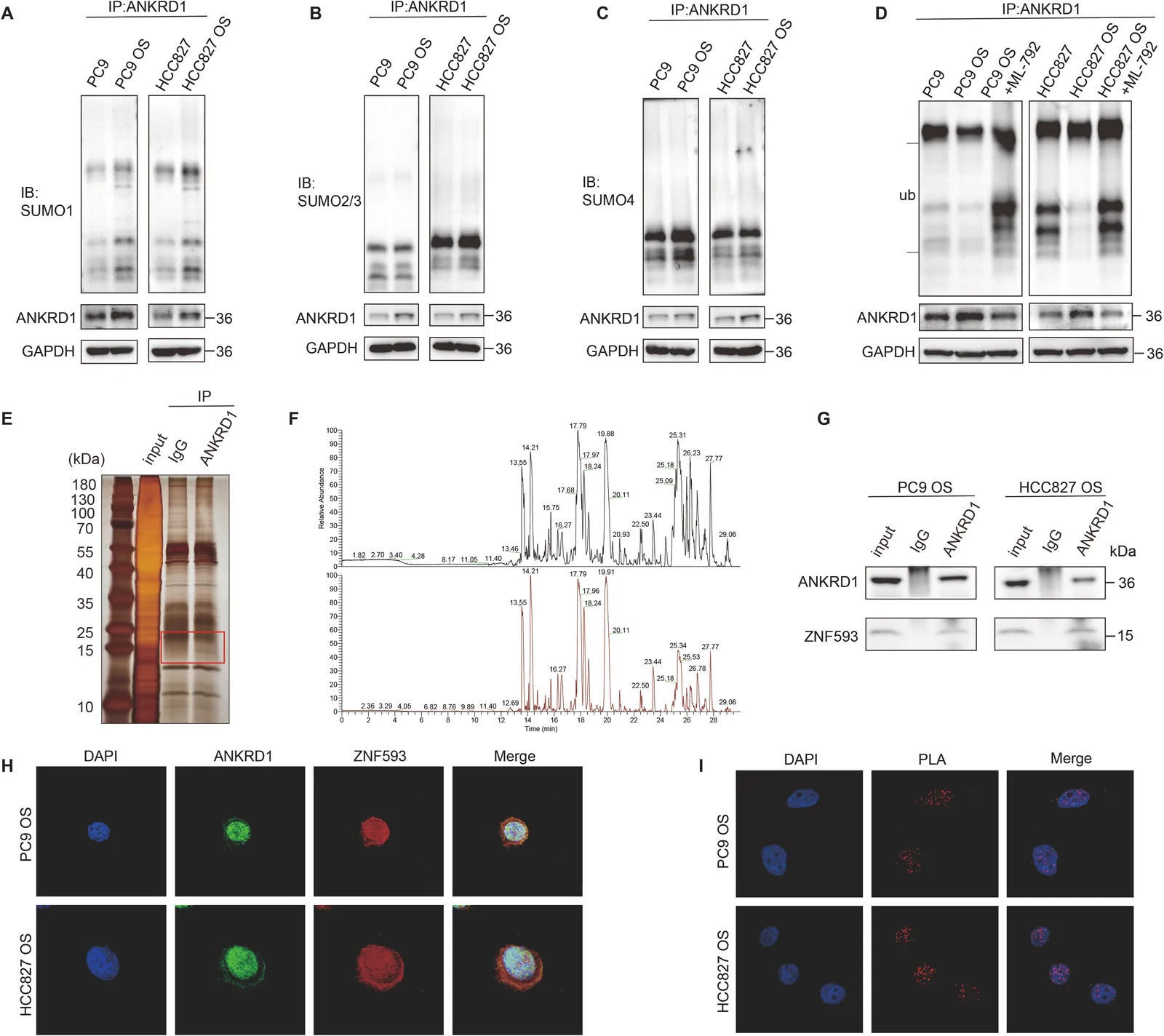
ANKRD1 undergoes SUMO1 modification and interacts with ZNF593. **(A)** Co-immunoprecipitation assay showing SUMO1 modification of ANKRD1. **(B)** Co-immunoprecipitation assay showing the absence of SUMO2/3 modification of ANKRD1. **(C)** Co-immunoprecipitation assay showing the absence of SUMO4 modification of ANKRD1. **(D)** Ubiquitination assay showing an inverse relationship between SUMOylation and ubiquitination of ANKRD1. **(E)** Mass spectrometry analysis identifying ZNF593 as a candidate ANKRD1 interactor. **(F)** ZNF593 among candidate binding partners. **(G)** Co-immunoprecipitation assay validating the interaction between ANKRD1 and ZNF593. **(H)** Immunofluorescence assay showing co-localization of ANKRD1 and ZNF593. **(I)** Proximity ligation assay confirming the interaction between ANKRD1 and ZNF593 *in situ*.

### 3.5 ZNF593 interacts with ANKRD1 and facilitates its SUMOylation-dependent stabilization

To identify upstream regulators of ANKRD1 SUMOylation, we performed mass spectrometry and identified ZNF593 as a candidate interactor (**Figures 4E, F**). This interaction was validated by Co-IP, IF co-localization, and PLA assays (**Figures 4G–I**), confirming a nuclear interaction. Functionally, ZNF593 depletion reduced SUMOylation while increasing ubiquitination of ANKRD1, leading to decreased protein stability (**Figures 5A–C**). CHX chase assays further demonstrated accelerated degradation of ANKRD1 upon ZNF593 knockdown, which could be rescued by proteasome inhibition (**Figure 5D**). Consistent with its role in maintaining ANKRD1 stability, ZNF593 knockdown phenocopied ANKRD1 depletion, including increased apoptosis, reduced proliferation, and suppression of MERTK–AKT signaling (**Figures 5E–J**). Together, these findings indicate that ZNF593 interacts with ANKRD1 and facilitates its SUMO1-dependent modification and stabilization; however, the precise biochemical mechanism by which ZNF593 promotes ANKRD1 SUMOylation remains to be determined.

**Figure 5:**
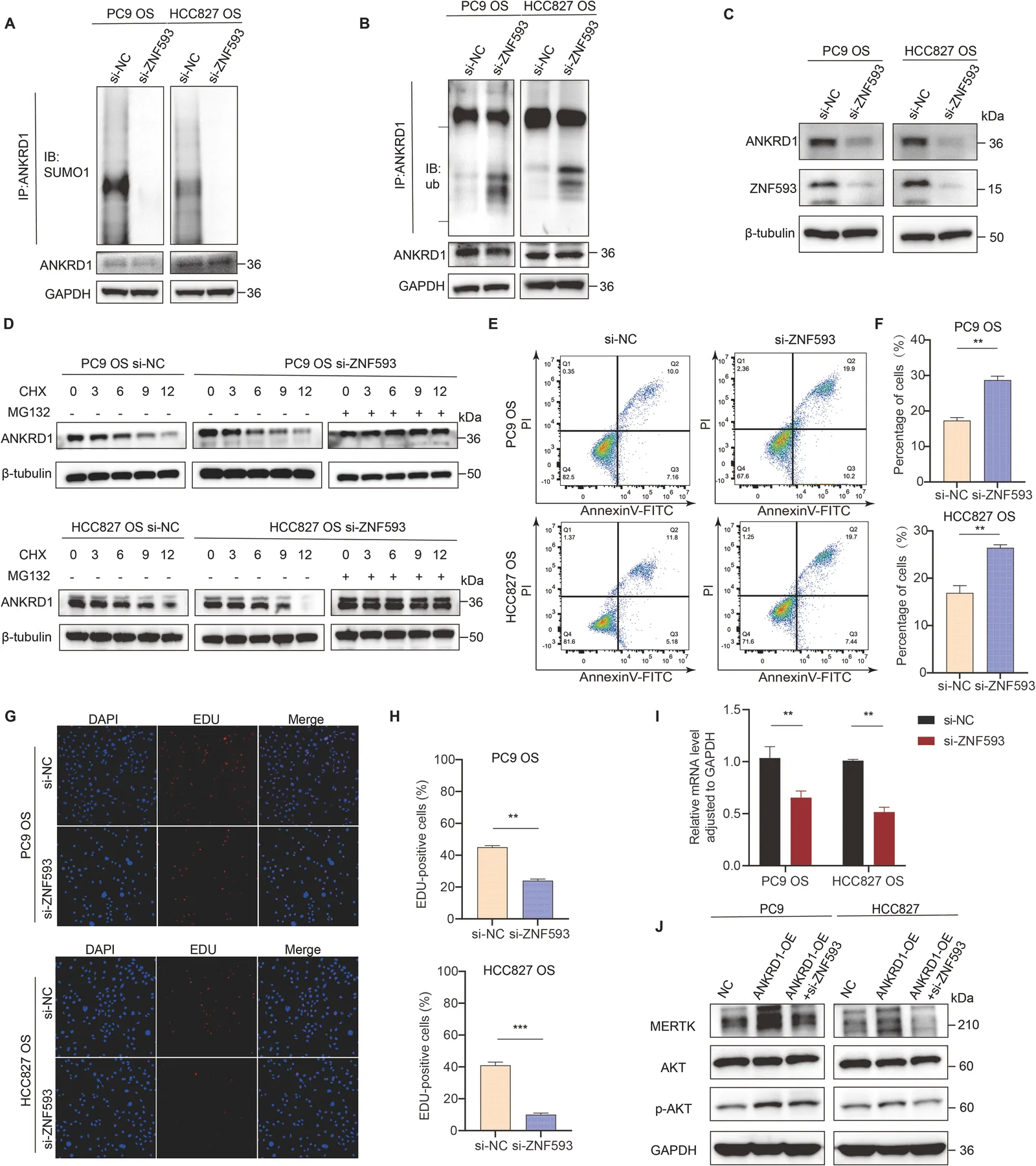
ZNF593 stabilizes ANKRD1 via SUMOylation-dependent inhibition of ubiquitination. **(A)** SUMOylation assay showing reduced SUMO1 modification of ANKRD1 upon ZNF593 depletion. **(B)** Ubiquitination assay showing increased ubiquitination of ANKRD1 following ZNF593 knockdown. **(C)** Western blot analysis showing decreased ANKRD1 protein levels upon ZNF593 depletion. **(D)** Cycloheximide chase assay showing accelerated degradation of ANKRD1 following ZNF593 knockdown, which was partially rescued by proteasome inhibition with MG132. **(E)** Representative flow cytometry plots of Annexin V/PI staining following ZNF593 knockdown. **(F)** Quantification of apoptotic cells corresponding to **(E)**. **(G)** Representative images of cell proliferation assays following ZNF593 knockdown. **(H)** Quantification of cell proliferation corresponding to **(G)**. **(I)** Western blot analysis showing reduced MERTK expression following ZNF593 depletion. **(J)** Western blot analysis showing decreased AKT phosphorylation following ZNF593 knockdown. Data are presented as mean ± SD from n = 3 independent experiments. Statistical significance was determined by Student’s *t*-test or one-way ANOVA as appropriate. *P* < 0.05 was considered statistically significant. **P < 0.01.

### 3.6 K41 coordinates ANKRD1 post-translational regulation and ZNF593 interaction

To dissect the structural basis of ANKRD1 regulation, we identified K5, K41, and K61 as candidate modification sites (**Figure 6A**). Among these, mutation of K41 uniquely disrupted both SUMOylation and ubiquitination (**Figures 6C, D**), identifying it as a key regulatory node coordinating these modifications. Notably, K41 mutation also impaired the interaction between ANKRD1 and ZNF593 (**Figure 6B**), consistent with structural prediction suggesting that K41 resides at the interaction interface (**Figure 6E**). Functionally, the K41 mutant failed to activate AKT signaling (**Figure 6F**) and exhibited reduced protein–protein interaction capacity (**Figure 6G**). In the orthotopic lung tumor model, K41 mutation markedly attenuated ANKRD1-driven tumor growth and resistance under osimertinib treatment (**Figure 6H**). Together, these findings identify K41 as a critical regulatory residue involved in ANKRD1 post-translational regulation and its interaction with ZNF593, thereby contributing to ANKRD1 stability and function.

**Figure 6:**
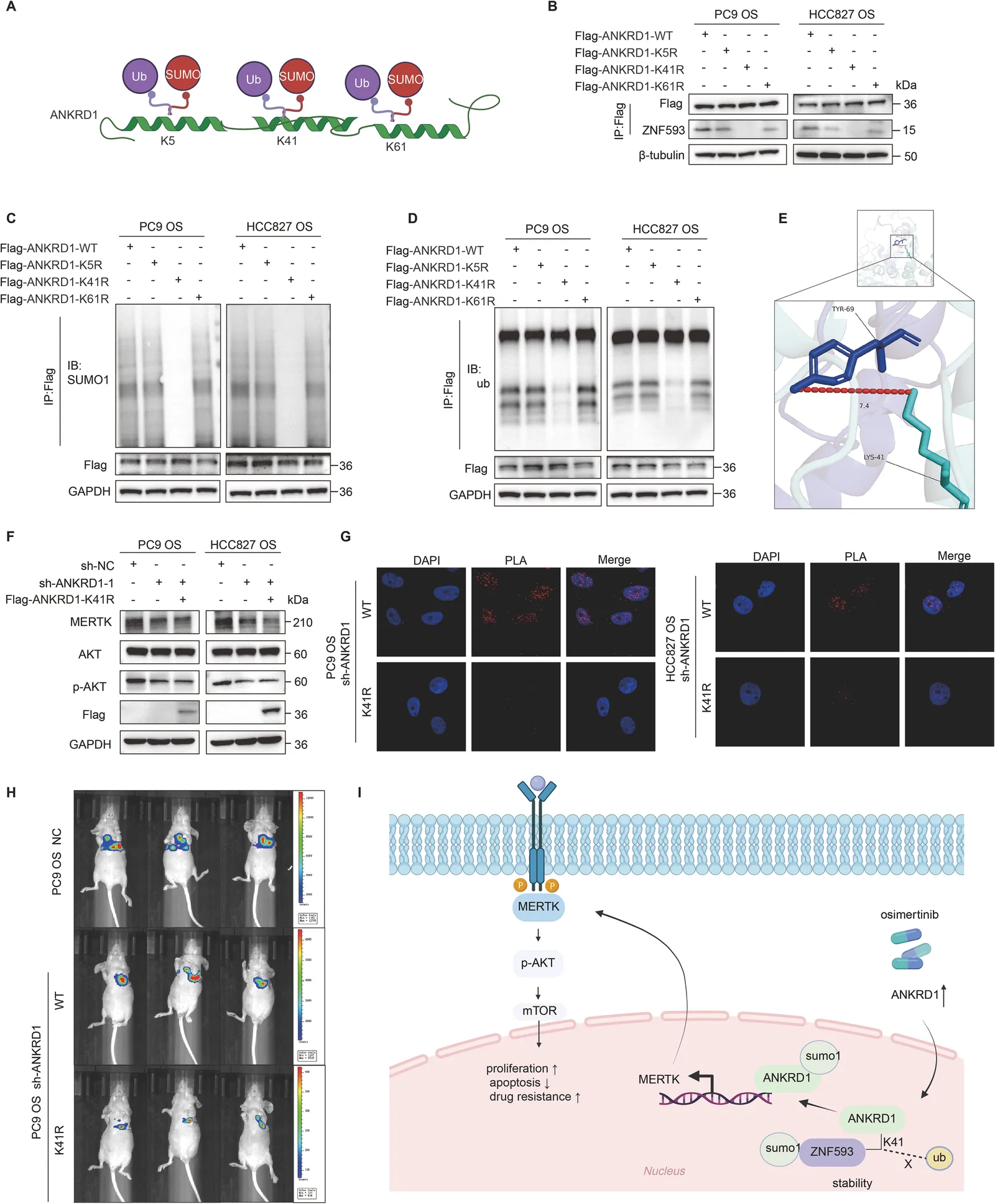
K41 coordinates ANKRD1 post-translational regulation and ZNF593 interaction. **(A)** Schematic diagram showing candidate lysine modification sites in ANKRD1. **(B)** Co-immunoprecipitation assay showing impaired interaction between ANKRD1 and ZNF593 upon K41 mutation. **(C)** SUMOylation assay showing reduced modification of ANKRD1 following K41 mutation. **(D)** Ubiquitination assay showing altered ubiquitination of ANKRD1 upon K41 mutation. **(E)** Structural prediction indicating localization of K41 at the interaction interface. **(F)** Western blot analysis showing impaired AKT activation in cells expressing the K41 mutant. **(G)** Proximity ligation assay showing reduced binding of the K41 mutant to interacting partners. **(H)** Representative bioluminescence images of the orthotopic lung tumor model under osimertinib treatment. **(I)** Schematic model of the SUMOylation–ANKRD1–MERTK regulatory axis in osimertinib resistance. Data are presented as mean ± SD from n = 3 independent experiments (*in vitro*) or n = 5 mice per group (*in vivo*). Statistical significance was determined by Student’s *t*-test or one-way ANOVA as appropriate. *P* < 0.05 was considered statistically significant.

## 4 Discussion

Although EGFR-mutant lung adenocarcinoma initially responds well to osimertinib, the emergence of acquired resistance is almost inevitable. Previous studies have largely attributed resistance to secondary EGFR mutations, bypass pathway activation, or lineage plasticity (21, 22). However, these models predominantly focus on localized alterations at the receptor or kinase level and fail to account for the systems-level adaptation, state stability, and reversibility observed under sustained therapeutic pressure. The concept of drug-tolerant persister cells further suggests that resistance is not solely driven by genetic alterations but instead reflects a dynamic cell-state transition orchestrated by transcriptional reprogramming and proteostasis remodeling (23, 24). Despite this progress, the upstream regulatory nodes that initiate and stabilize this adaptive transition remain poorly defined.

Our findings support a mechanism in which proteostasis-dependent regulation of ANKRD1 contributes to acquired osimertinib resistance in EGFR-mutant LUAD. ZNF593 interacts with ANKRD1 and facilitates its SUMO1-dependent modification, thereby supporting ANKRD1 protein stability. Stabilized ANKRD1 subsequently promotes MERTK expression and sustains downstream AKT signaling. These findings connect post-translational regulation of a stress-responsive protein with receptor tyrosine kinase-mediated survival signaling and provide a mechanistic explanation for how tumor cells may maintain survival under sustained osimertinib pressure.

This mechanism complements established models of EGFR-TKI resistance involving secondary EGFR alterations, bypass pathway activation, and phenotypic plasticity (25). Rather than representing an alternative to these mechanisms, our findings suggest that stress-associated regulation of protein stability may provide an additional layer through which survival signaling is reactivated during therapeutic pressure (26). In this context, stabilization of ANKRD1 may facilitate transcriptional and signaling adaptations that contribute to the transition toward a resistant state. However, whether this mechanism operates broadly across distinct resistance states or genetic backgrounds remains to be determined.

At the downstream signaling level, our data identify MERTK–AKT signaling as an important component of ANKRD1-mediated survival. MERTK has previously been implicated in tumor cell survival, immune regulation, and therapeutic resistance across multiple cancer types (27). Our data support ANKRD1 as a transcriptional activator of MERTK, thereby directly coupling stress-responsive transcriptional programs to RTK signaling output (28). Importantly, re-expression of MERTK only partially restored AKT activation and the cellular phenotypes caused by ANKRD1 depletion, indicating that MERTK does not fully account for the biological effects of ANKRD1. Thus, the ANKRD1–MERTK–AKT axis should be considered an important, but not exclusive, downstream mechanism of ANKRD1-mediated osimertinib resistance. Additional MERTK-independent transcriptional or signaling programs may also contribute and warrant further investigation.

At the post-translational level, our results indicate that ANKRD1 stability is regulated by the balance between SUMOylation and ubiquitination. In contrast to phosphorylation-mediated rapid signaling responses (29), SUMOylation can regulate protein stability, localization, and protein–protein interactions, providing a potential mechanism for maintaining stress-adaptive protein states (30). We found that ZNF593 physically interacts with ANKRD1 and that depletion of ZNF593 reduces ANKRD1 SUMOylation, increases its ubiquitination, and accelerates proteasomal degradation. These observations support a role for ZNF593 in facilitating ANKRD1 SUMO1 modification and stabilization. However, our current evidence does not establish ZNF593 as a bona fide SUMO E3 ligase. ZNF593 may instead facilitate the recruitment or accessibility of the SUMO conjugation machinery to ANKRD1, potentially involving UBC9. The precise biochemical mechanism underlying ZNF593-facilitated ANKRD1 SUMOylation therefore remains to be determined.

K41 emerged as a particularly important residue in this regulatory process. Substitution of K41 markedly reduced both SUMOylation and ubiquitination of ANKRD1, which is compatible with the possibility that K41 serves as a shared lysine subject to competing post-translational modifications. Because SUMO and ubiquitin are both covalently conjugated to lysine residues, modification of K41 by one pathway could potentially influence its accessibility to the other. Notably, however, K41R also weakened the interaction between ANKRD1 and ZNF593, suggesting that K41 may additionally contribute to the ANKRD1–ZNF593 interaction interface or maintain a local structural environment required for efficient post-translational regulation. These possibilities are not mutually exclusive. We therefore propose that K41 may have a dual regulatory role, coordinating modification-dependent proteostasis while also contributing to protein–protein interaction.

The functional importance of K41 is further supported by the impaired AKT activation and attenuated tumor growth and resistance phenotypes observed with the K41 mutant. These findings indicate that K41-dependent regulation is important for ANKRD1 function under osimertinib pressure. However, rather than defining K41 as a definitive “molecular switch,” our data support its role as a critical regulatory residue within the ANKRD1 proteostatic network. Whether K41 also influences additional ANKRD1-interacting proteins or broader signaling programs remains an important question for future studies.

From a translational perspective, these findings generate several hypotheses that warrant further investigation. The increase in ANKRD1 expression associated with osimertinib resistance suggests that ANKRD1 could be evaluated as a candidate biomarker in larger, clinically annotated cohorts. Similarly, the functional involvement of MERTK–AKT signaling provides a rationale for testing whether pharmacological inhibition of MERTK can enhance osimertinib sensitivity in ANKRD1-high resistant tumors. The K41-dependent regulation of ANKRD1 also raises the possibility that the ANKRD1–ZNF593 interaction or associated proteostatic machinery could represent a pharmacologically exploitable vulnerability. However, these possibilities were not directly tested in the present study and should therefore be considered potential directions for future preclinical and translational investigation rather than established therapeutic strategies.

Several limitations of this study should be acknowledged. First, clinical validation was limited to three paired tumor specimens obtained before osimertinib treatment and after the development of acquired resistance. Although these paired samples provided preliminary clinical support for increased ANKRD1 expression following resistance, the limited sample size precluded robust assessment of associations with treatment response or survival, and validation in larger, clinically annotated cohorts is required. Second, although ZNF593 facilitates ANKRD1 SUMOylation, its precise biochemical role and relationship with the SUMO conjugation machinery remain unresolved. Third, while the K41R experiments support a critical role for K41 in ANKRD1 post-translational regulation, direct assignment of K41 as a SUMO or ubiquitin acceptor site will require additional biochemical or mass-spectrometric evidence. Finally, the *in vivo* experiments were performed in immunodeficient nude mice. Given the known immunoregulatory functions of MERTK, these models primarily address tumor cell-intrinsic effects and cannot fully evaluate the contribution of the immune microenvironment. Future studies using immunocompetent or humanized models will be required to investigate this aspect.

## 5 Conclusion

In summary, our study identifies a proteostasis-related mechanism contributing to acquired osimertinib resistance in EGFR-mutant LUAD. ZNF593 interacts with ANKRD1 and facilitates its SUMO1-dependent modification, supporting ANKRD1 stability, while stabilized ANKRD1 promotes MERTK–AKT signaling and tumor cell survival under therapeutic pressure (**Figure 6I**). K41 appears to be a critical regulatory residue linking ANKRD1 post-translational regulation with ZNF593 interaction. Together, these findings provide mechanistic insight into the regulation of ANKRD1 during osimertinib resistance and provide a rationale for further investigation of the ZNF593–ANKRD1–MERTK axis in resistant LUAD.

## Data Availability

The original contributions presented in the study are included in the article/**Supplementary Material**. Further inquiries can be directed to the corresponding authors.
