# Supplementary material for: SUMOylation counteracts ubiquitination to stabilize ANKRD1 and promote osimertinib resistance in lung adenocarcinoma
Source: Front Oncol. 2026 Oct 22;16:1960448. doi: 10.3389/fonc.2026.1960448 (PMC13735745; doi:10.3389/fonc.2026.1960448)
Supplement: Supplementary file 1 [file Supplementaryfile1.docx]

| **Target Protein** | **Host Species** | **Catalog Number** | **Supplier** |
| --- | --- | --- | --- |
| ANKRD1 | Rabbit | 11427-1-AP | Proteintech (USA) |
| ANKRD1 | Mouse | 67775-1-Ig | Proteintech (USA) |
| MERTK | Rabbit | #4319 | Cell Signaling Technology (USA) |
| AKT | Rabbit | #4691 | Cell Signaling Technology (USA) |
| Phospho-AKT (Ser473) | Rabbit | #4060 | Cell Signaling Technology (USA) |
| ZNF593 | Rabbit | 19426-1-AP | Proteintech (USA) |
| β-Tubulin | Mouse | 10094-1-AP | Proteintech (USA) |
| SUMO1 | Rabbit | 10329-1-AP | Proteintech (USA) |
| SUMO2/3 | Rabbit | 11251-1-AP | Proteintech (USA) |
| SUMO4 | Rabbit | A9016 | ABclonal (China) |
| Ubiquitin | Rabbit | 10201-2-AP | Proteintech (USA) |
| GAPDH | Mouse | 10494-1-AP | Proteintech (USA) |

**Supplementary Fig. S1. Functional validation of ANKRD1 overexpression and its regulation of MERTK–AKT signaling.**

(A) Western blot analysis confirming ANKRD1 overexpression efficiency in LUAD cells.

(B) Cell viability assays showing increased IC₅₀ of osimertinib upon ANKRD1 overexpression.

(C) EdU incorporation assays showing enhanced cell proliferation following ANKRD1 overexpression.

(D) Quantification of EdU-positive cells corresponding to (C).

(E) Flow cytometry analysis of apoptosis showing reduced apoptosis upon ANKRD1 overexpression.

(F) Quantification of apoptotic cells corresponding to (E).

(G) Dual-luciferase reporter assay showing transcriptional activation of MERTK promoter activity by ANKRD1 overexpression.

(H) Western blot analysis showing increased MERTK expression and AKT phosphorylation levels in ANKRD1-overexpressing cells. Pharmacological inhibition of MERTK reduced AKT phosphorylation.

Data are presented as mean ± SEM from n = 3 independent experiments. Statistical significance was determined by Student’s *t*-test or one-way ANOVA as appropriate. *P* < 0.05 was considered statistically significant.
